# Treatment of Hepatic Epithelioid Hemangioendothelioma: Finding Uses for Thalidomide in a New Era of Medicine

**DOI:** 10.1155/2015/326795

**Published:** 2015-06-17

**Authors:** Matthew P. Soape, Rashmi Verma, J. Drew Payne, Mitchell Wachtel, Fred Hardwicke, Everardo Cobos

**Affiliations:** Texas Tech University Health Sciences Center, 3601 4th Street, Lubbock, TX 79430, USA

## Abstract

Hepatic epithelioid hemangioendothelioma (HEH) is extremely rare, occurring in 1 to 2 per 100,000, with chemotherapy options not well defined. Our case involved a 49-year-old female who had hepatic masses and metastasis to the lungs with a liver biopsy revealing HEH. After developing a rash from sorafenib, thalidomide was started with the progression of disease stabilized. Resection is only an option in 10% of the cases; therefore, chemotherapy is the only line of treatment. Newer chemotherapy alternatives are targeting angiogenesis via the vascular endothelial growth factor. Thalidomide was first used as an antiemetic, but, sadly, soon linked to phocomelia birth defects. Given the mechanism of action against angiogenesis, thalidomide has a valid role in vascular tumors. In conclusion, the use of thalidomide as chemotherapy is novel and promising, especially in the setting of a rare vascular liver tumor such as HEH.

## 1. Introduction

Vascular in origin, hepatic epithelioid hemangioendothelioma (HEH) is an extremely rare diagnosis; therefore, treatment for this malignancy has not been well defined. Much of the data on HEH are in case studies and small case series with treatment usually confined to only a few options. Given the vascular nature of this cancer, thalidomide has been used with some degree of success. In the mid-twentieth century, thalidomide was used for morning sickness in pregnant women, and their babies developed well-documented phocomelia birth defects.

From those horrific malformations, continued investigation into the explanation of thalidomide involvement has led to a better understanding of its harmful effects on angiogenesis. With this discovery, the careful use of the medication has been employed as chemotherapy in cancers such as HEH. In this case report, we present a female diagnosed with metastatic HEH who has been successfully treated with thalidomide chemotherapy.

## 2. Case Report

We present a 49-year-old Hispanic female originally seen in the ER for atypical chest pain needing cardiac evaluation. Patient presented months back with chest pain that started early the morning of admission, intermittent in nature, worsened with deep inspiration, and radiated to her back. No abdominal pain was noted, but she did complain of associated nausea, diaphoresis, fatigue, and dizziness. She also reported 30-pound weight loss in the last month with no change in appetite discussed. Past medical history was only significant for type 2 diabetes mellitus. Family history was significant for coronary artery disease in her father and stomach cancer in her mother. No pertinent surgical history was given. She denied any tobacco, alcohol, and illicit drug use.

**Figure 1:**
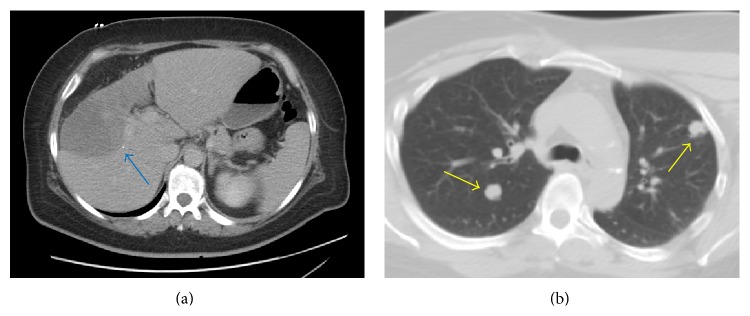
(a) Computed tomography (CT) of the abdomen showing a large hepatic mass, measuring 12 cm × 5.5 cm and designated by the blue arrow. (b) CT of the chest showing metastatic nodules, designated by yellow arrows.

Full cardiac evaluation, including EKG, cardiac marker testing, and chest X-ray, was negative. Upon further review of her chart, 3 years prior to this presentation, a computed tomography (CT) of the abdomen and pelvis was ordered by an urgent care physician secondary to abdominal pain. It showed multiple hypodense masses in the right and left lobe of the liver. The largest one showed a lobulated margin measuring 6.7 cm × 5.5 cm. The masses did not show typical characteristics of hemangioma or simple hepatic cyst. Also noted were fatty liver changes. There was no older study for comparison at that time. A short term repeat CT scan was recommended but she was lost to follow-up. During this admission, CT abdomen with intravenous contrast showed hepatic masses, largest being 12 cm × 5.5 cm, and development of small pulmonary nodules worrisome for metastasis (Figure 1). Small lucencies within the thoracolumbar spine were also found and thought to be focal areas of osteopenia, but metastasis could not be excluded.

**Figure 2:**
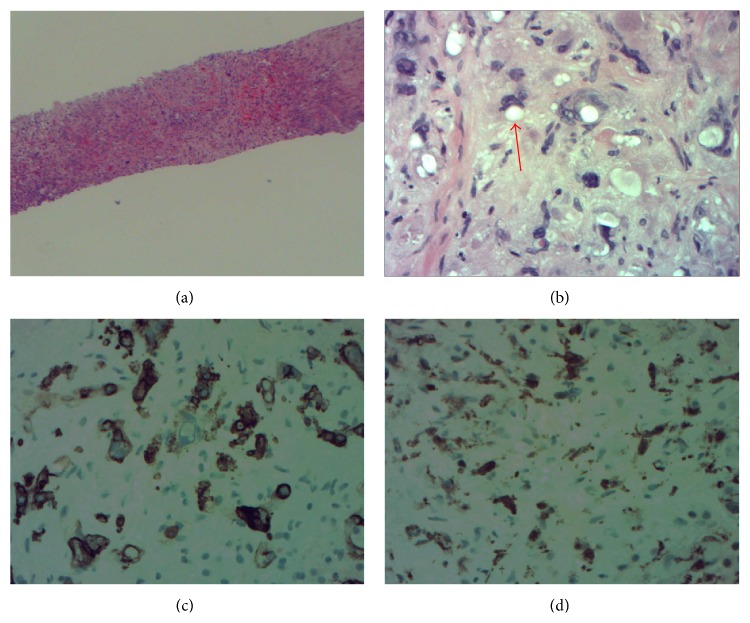
(a) Replacing hepatocytes is tumor with sometimes large, often spindled, aberrant nuclei amid abundant light pink, feathery matrixes (H&E, 100x). (b) Uniformly dark, cancerous nuclei, sometimes huge, lie in variably colored cytoplasm, often not evident. Cancer cells lie in isolation and in epithelioid groups. At the arrowhead, a group lies about a vacuole, which is a vascular lumen (H&E, 400x). (c) Cancer cell cytoplasm often expresses CD34, which emphasizes the epithelioid pattern (DAB, 400x). (d) Many cancer cell nuclei, often juxtaposed to vacuoles, express Factor XIIIa (DAB, 400x). Gold bars are 200 *μ* long.

Initial gastrointestinal evaluation revealed normal bilirubin, PT, PTT, and INR. Hepatitis B and hepatitis C were both negative. She did exhibit elevated alkaline phosphatase of 311 IU/L and elevated cancer markers, including CEA of 6.0 ng/mL, CA 19-9 of 74.8 units/mL, and CA 125 of 36.3 units/mL. Ultrasound guided liver biopsy was elected at this time and pathology was consistent with HEH (Figure 2). Oncology was then consulted and surgical resection was deferred in the setting of metastatic disease to the lungs. She was started on the oral multikinase inhibitor sorafenib (Nexavar) but soon developed a worsening rash. Thalidomide 200 mg nightly was started in its place with the patient tolerating therapy for the past 10 months. One year after her diagnosis, a repeat CT scan of the abdomen showed increased size of the right and left lobe pulmonary mass lesions, ranging from 4 mm to 7 mm. The largest mass in the liver had also increased to a size of 13.2 cm × 7.1 cm. However, her laboratory values including bilirubin and alkaline phosphatase have been stable; therefore, thalidomide has been continued.

## 3. Discussion 

The term epithelioid hemangioma was coined by Weiss and Enzinger in 1982 to describe a distinct entity, a soft tissue vascular tumor of endothelial origin with a clinical course intermediate between benign hemangioma and malignant angiosarcoma [1]. This malignant hemangioma is extremely rare, occurring in only <1 to 2 per 100,000 patients with a female to male sex ratio of 1.5 to 1 [2]. Presentation is commonly asymptomatic, as in our patient, where she was evaluated for chest pain with no reported abdominal pain. 87% of patients presented with bilobar and multifocal disease, and 37% of patients presented with extrahepatic involvement [3]. Both multifocal and extrahepatic involvements were observed in our patient. HEH is usually a low grade malignant tumor with a slow progression phenotype. HEH, in contrast to many other types of primary liver tumor, does not arise in a background of chronic liver disease. Speculated risk factors include oral contraceptives, vinyl chloride, and hepatitis B infection [4], none of which was admitted by the patient, and hepatitis panel was negative. Histology is characterized as vascular endothelium in origin. Histology shows nests and cords of spindle to epithelioid cells embedded in a hyaline and myxoid stroma with closer examination of cells having prominent cytoplasmic vacuoles with a characteristic signet ring formation [5]. Immunohistochemical stains are positive for endothelial markers such as CD31, CD34, and Factor XIII related antigen [5]. Our case showed positive stains for both CD34 and Factor XIII related antigen.

In regard to treatment, resection is preferable with a 75% 5-year survival rate. However, resection is usually only an option in 10% of the cases [3]. Therefore, chemotherapy is the only available option in most cases. Other procedure treatments for HEH are limited to liver-liver transplant and transcatheter arterial chemoembolization (TACE). As in our case, metastatic disease had already been confirmed with pulmonary involvement, making resection not a viable option. In the past, chemotherapy agents employed have been doxorubicin, vincristine, interferon-alpha, 5-FU, and thalidomide. A newer chemotherapy alternative includes vascular endothelial growth factor (VEGF) targeted therapy. Angiogenesis in malignancy relies on VEGF and fibroblast growth factors (FGFs). Angiogenesis and malignancy association has numerous proposed mechanisms but the most accepted is an overexpression of VEGF [6].

In 1957, thalidomide was first released in West Germany. It was intended as a hypnotic and sedative medication but soon found favor working against nausea in morning sickness during pregnancy. Common side effects include somnolence, edema, hypotension, myelosuppression, peripheral neuritis, interstitial lung disease, and pneumonia. Sadly, thalidomide was also discovered to have a high association with birth defects such as phocomelia, a malformation of limbs. The phocomelia was severe with formation of the limbs appearing to be stunted and underdeveloped. 10,000 children were thought to be affected before it was taken off the market. One proposed teratogenic mechanism includes halting of angiogenesis by inhibition of fibroblastic growth factor (bFGF) and/or VEGF, which has been verified with in vivo studies [7]. Cereblon inactivation has also been suggested as a possible mechanism. This protein has a positive regulatory effect via ubiquitination on FGF8 and FGF10. FGF8 has a direct effect on limb formation. As indicated in a 2009 study, limb formation is thought to be highly susceptible due to the limb's “relatively immature, highly angiogenic vessel network” [8]. Thalidomide has been shown to be a well-tolerated medication. Two recent case reports of patients diagnosed with HEH demonstrate thalidomide tolerance factor, controlling the disease progression over an extended amount of time (as long as 109 months) [9, 10].

Other than its effects on vascular growth, thalidomide also inhibits tumor necrosis factor (TNF)-*α*, various interleukins (IL-6, IL-10, and IL-12) production, and NF-*κ*B and COX-2 activity. It modulates the production of IFN-*γ* and enhances the production of IL-2, IL-4, and IL-5 by immune cells. It also increases lymphocyte count, costimulates T cells, and modulates natural killer cell cytotoxicity [11]. Due to these regulatory factors, thalidomide has been recently used to treat multiple myeloma and erythema nodosum leprosum. It is also being investigated in therapeutic strategy for vascular malformations and hereditary hemorrhagic telangiectasia [12].

In conclusion, both the diagnosis of HEH and the use of thalidomide in a vascular mediated tumor are extremely rare. Given the mechanism of action against angiogenesis, thalidomide does show some benefit in treatment for such cancers. Furthermore, given the direction of cell receptor and/or growth factor guided chemotherapy, thalidomide may hold promising future derivatives that can better target malignant hepatic hemangiomas and other malignancies.

## References

[1] Weiss S. W., Enzinger F. M. (1982). Epitheloid hemangioendothelioma: a vascular tumor often mistaken for a carcinoma. *Cancer*.

[2] Taal B. G., Visser O. (2004). Epidemiology of neuroendocrine tumours. *Neuroendocrinology*.

[3] Mehrabi A., Kashfi A., Fonouni H. (2006). Primary malignant hepatic epithelioid hemangioendothelioma: a comprehensive review of the literature with emphasis on the surgical therapy. *Cancer*.

[4] Grossman E. J., Millis J. M. (2010). Liver transplantation for non-hepatocellular carcinoma malignancy: indications, limitations, and analysis of the current literature. *Liver Transplantation*.

[5] Makhlouf H. R., Ishak K. G., Goodman Z. D. (1999). Epithelioid hemangioendothelioma of the liver: a clinicopathologic study of 137 cases. *Cancer*.

[6] Scappaticci F. A. (2002). Mechanisms and future directions for angiogenesis-based cancer therapies. *Journal of Clinical Oncology*.

[7] D'Amato R. J., Loughnan M. S., Flynn E., Folkman J. (1994). Thalidomide is an inhibitor of angiogenesis. *Proceedings of the National Academy of Sciences of the United States of America*.

[8] Therapontos C., Erskine L., Gardner E. R., Figg W. D., Vargesson N. (2009). Thalidomide induces limb defects by preventing angiogenic outgrowth during early limb formation. *Proceedings of the National Academy of Sciences of the United States of America*.

[9] Raphael C., Hudson E., Williams L., Lester J. F., Savage P. M. (2010). Successful treatment of metastatic hepatic epithelioid hemangioendothelioma with thalidomide: a case report. *Journal of Medical Case Reports*.

[10] Salech F., Valderrama S., Nervi B. (2011). Thalidomide for the treatment of metastatic hepatic epithelioid hemangioendothelioma: a case report with a long term follow-up. *Annals of Hepatology*.

[11] Singhal S., Mehta J. (2002). Thalidomide in cancer. *Biomedicine & Pharmacotherapy*.

[12] Lebrin F., Srun S., Raymond K. (2010). Thalidomide stimulates vessel maturation and reduces epistaxis in individuals with hereditary hemorrhagic telangiectasia. *Nature Medicine*.

